# Supplementary material for: A multi-omics analysis unveils functional and regulatory links between hydroxybenzene and aromatic amino acid metabolism in Candida albicans
Source: mSystems. 2025 Oct 8;10(11):e00226-25. doi: 10.1128/msystems.00226-25 (PMC12625712; doi:10.1128/msystems.00226-25)
Supplement: Supplemental figures — Figures S1 to S4. [file msystems.00226-25-s0001.docx]

**
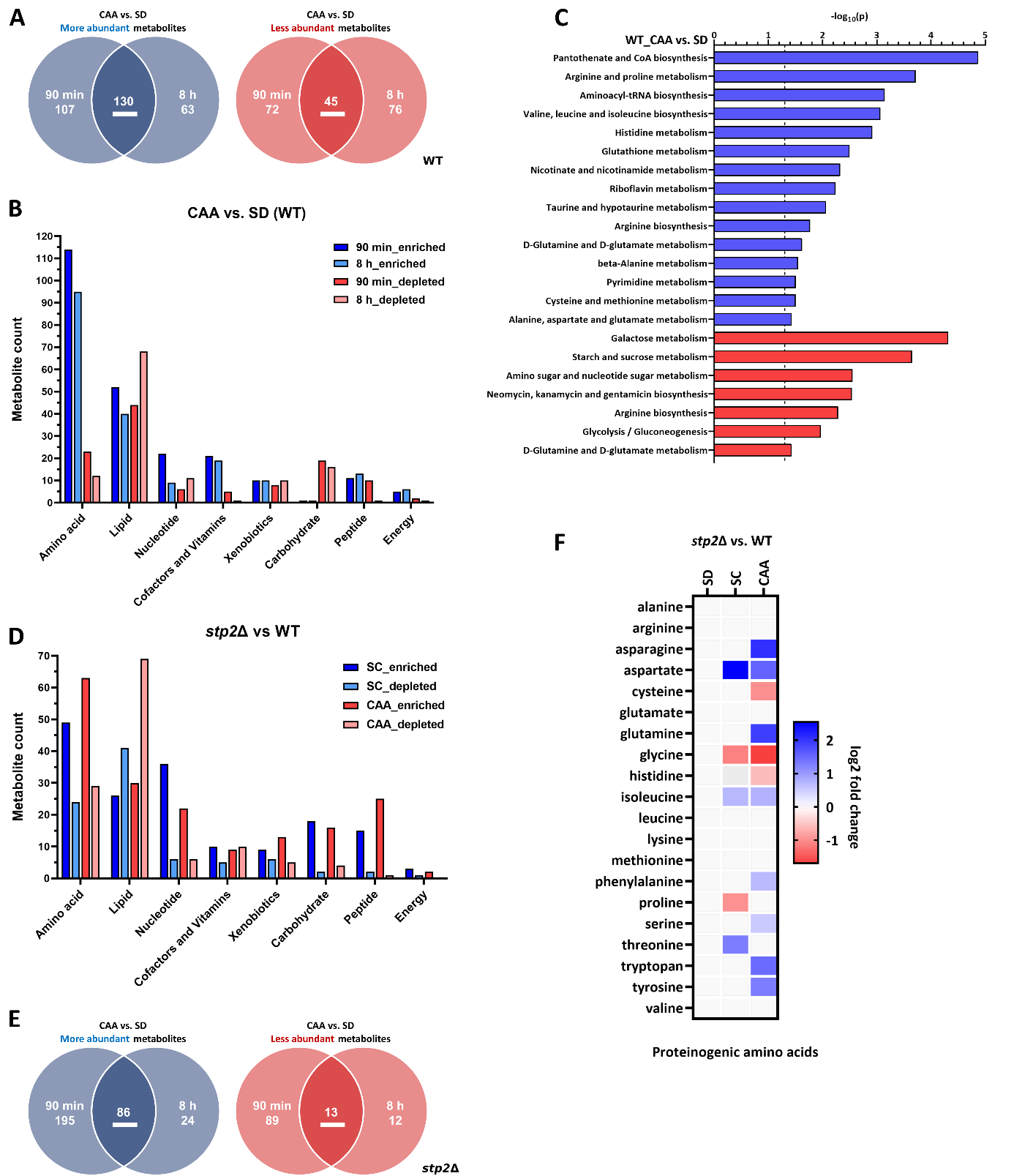
**

**Fig. S1: Metabolic changes in the WT and *stp2*Δ in response to amino acid-rich conditions**

**A**) Metabolites with significantly increased or lowered abundance (p<0.05) in CAA compared to SD in the WT after 90 min and 8 h incubation. **B**) Number of significantly more or less abundant metabolites (p<0.5) in the WT after incubation in CAA for 90 min and 8 h compared to SD. Metabolites are grouped by their respective “Super pathways”. **C**) Metabolites either significantly more (blue) or less (red) abundant in CAA vs. SD in the WT possessing a unique HMDB identifier were used for KEGG pathway enrichment analysis via “MetaboAnalyst”. Dashed line indicates the significance threshold of p=-log10(0.05). **D**) Number of significantly more or less abundant metabolites (p<0.05) in *stp2*Δ vs. WT in SC and CAA grouped by their respective “Super pathways”. **E**) Metabolites with significantly increased or lowered abundance (p<0.05) in CAA compared to SD in *stp2*Δ after 90 min and 8 h incubation. **F**) Abundance changes (log2(fold change)) of intracellular amino acids in *stp2*Δ vs. WT in the indicated conditions. Changes not meeting the significance threshold (p<0.05) are shown in grey.


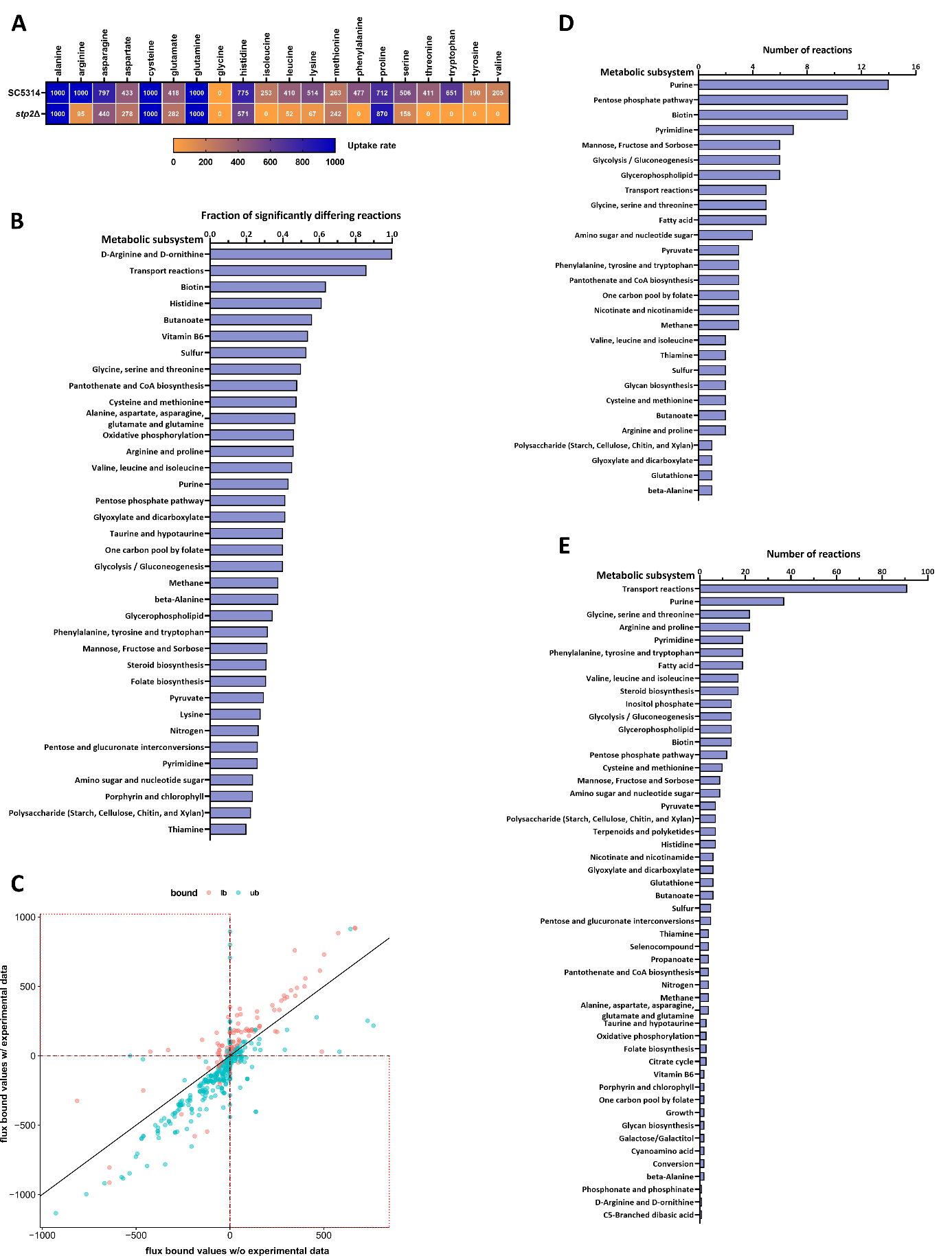


**Fig. S2: Comparison of FVA for WT and *stp2*Δ in amino acid-free and rich medium with and without experimentally obtained metabolome data**

**A**) Uptake rates for single amino acids used for the FVA. Rates were adapted from mass spectrometry measurements, comparing spent media from *C. albicans* cultures to medium reference to calculate log2(fold changes) for each amino acid. Greatest fold change was observed for arginine in the WT and set to maximal uptake rate of 1000. Remaining rates were calculated relative to this maximum or set to 0, if showing no significant difference from the blank. Alanine, cysteine and glutamine displayed possible secretion by the fungus, therefore their uptake was not constraint. **B**) Metabolic reactions carrying significantly (p<0.05) different metabolic flux in *stp2*Δ vs. WT in SC as fractions of their respective metabolic subsystems in FVA without experimental data. **C**) Scatter plot showing absolute flux difference between *stp2*Δ vs. WT in SC for FVA without and with experimental metabolome data. Per reaction only the higher of lb or ub is indicated. Diagonal line indicates equal flux differences in both analyses. Pearson correlation for all fluxes is 0.878. Reactions located in the quadrants framed with dashed red lines indicate reaction with opposing directionalities in both analyses. **D**) Number of reactions with opposing directionalities assigned to their respective metabolic subsystem. **E**) Number of reactions per metabolic subsystem with significant differences (p<0.05) in flux difference between *stp2*Δ vs. WT in SC for FVA without and with experimental metabolome data. Only reactions with absolute log2(fold change)≥1 in either lb or ub were included.


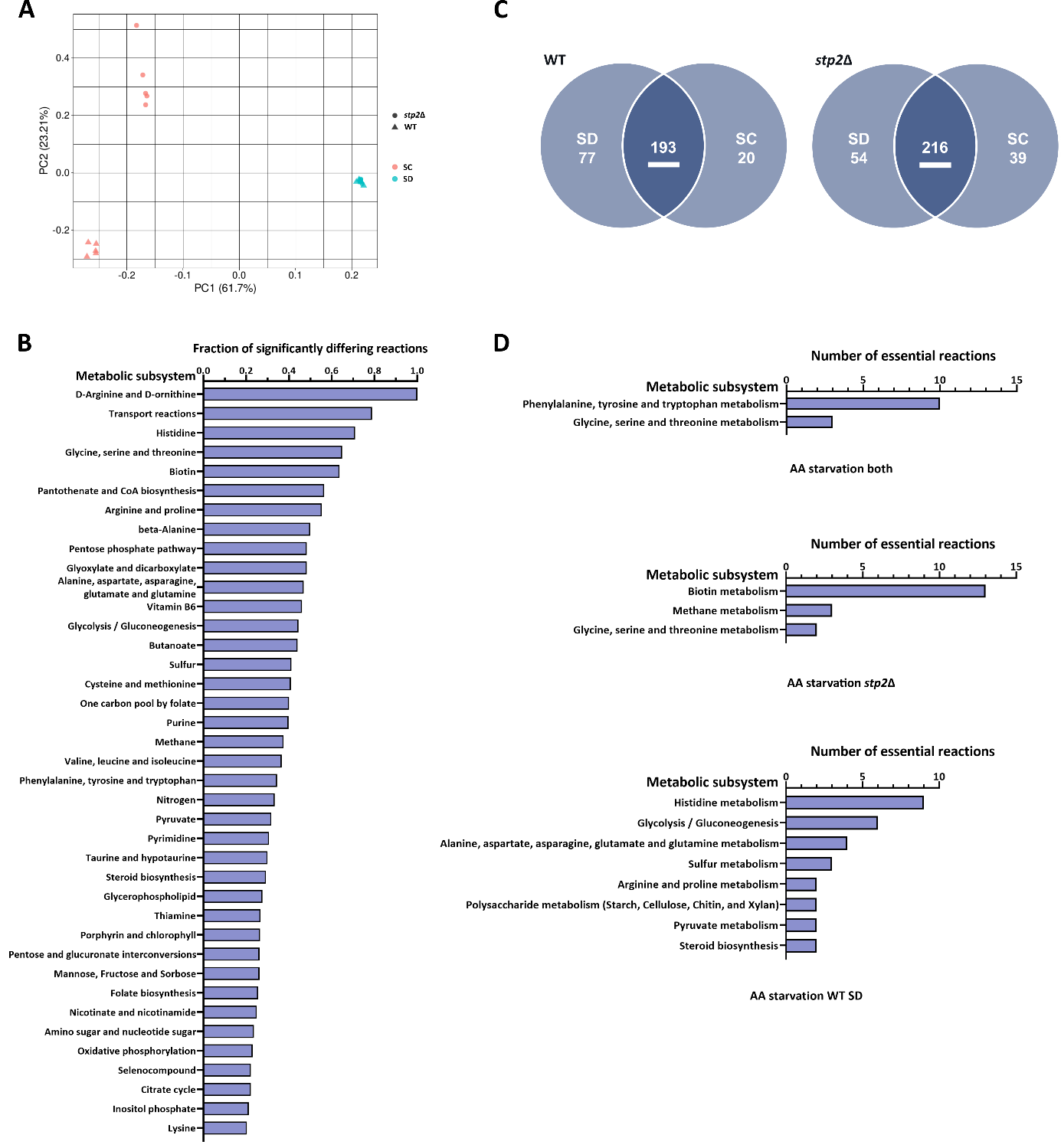


**Fig. S3: Flux differences and essential reactions in *stp2*Δ in amino acid-rich medium**

**A**) PCA of FVA-simulated metabolic flux of WT and *stp2*Δ in SD and SC. **B**) Metabolic reactions carrying significantly (p<0.05) different metabolic flux in *stp2*Δ vs. WT in SC as fractions of their respective metabolic subsystems. **C**) Comparison of reactions predicted to be essential in SD and SC for WT and *stp2*Δ. **D**) Number of essential reactions specifically for conditions of amino acid starvation (*stp2*Δ in SC and WT in SD). Only subsystems with at least two essential reactions are displayed for: Reactions essential in *stp2*Δ in SC and WT in SD; in *stp2*Δ in SC but not in WT in SD; essential in WT in SD but not in *stp2*Δ in SC.


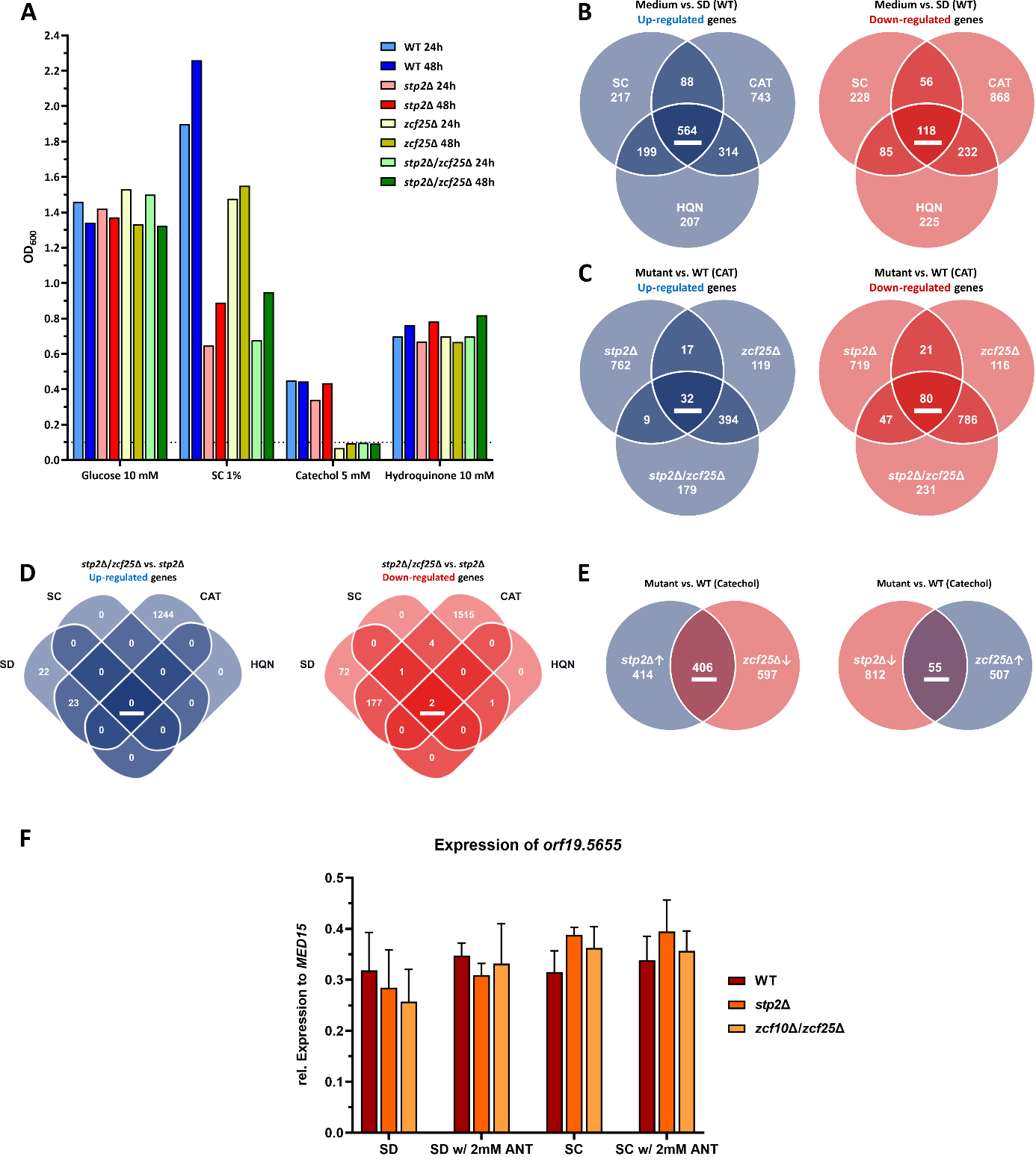


**Fig. S4: Zcf25 and Stp2 mediate regulatory connections of the 3OAP and SHKP**

**A**) OD_600_ of indicated *C. albicans* strains in liquid media and conditions used for the RNA-Seq with the extended incubation time to 24 h or 48 h at 30°C (Biological unicates). Media contained only the indicated C-source and was supplemented with YNB and 0.5% ammonium sulfate. **B**) Significantly up-or down-regulated genes (p>0.05; absolute log2(fold change)≥1) in the WT in the indicated conditions compared to SD. **C**) Up-or down-regulated genes in *stp2*Δ, *zcf25*Δ and *stp2*Δ/*zcf25*Δ vs. WT in response to catechol. **D**) Significantly up-or down-regulated genes in *stp2*Δ/*zcf25*Δ vs. *stp2*Δ in response to the indicated media. **E**) Overlay of up-regulated genes in *stp2*Δ vs. WT and down-regulated in *zcf25*Δ vs. WT and vice versa. **F**) Expression of *orf19.5655* relative to *MED15* in SD and SC medium with and without 2 mM anthranilate (ANT) in the indicated *C. albicans* strains. Expression levels were determined in technical and biological triplicates.
